# A shared Runx1-bound *Zbtb16* enhancer directs innate and innate-like lymphoid lineage development

**DOI:** 10.1038/s41467-017-00882-0

**Published:** 2017-10-16

**Authors:** Ai-Ping Mao, Isabel E. Ishizuka, Darshan N. Kasal, Malay Mandal, Albert Bendelac

**Affiliations:** 1 Committee on Immunology, University of Chicago, Chicago, IL 60637 USA; 2 Department of Pathology, University of Chicago, Chicago, IL 60637 USA; 3 Department of Medicine, Section of Rheumatology and Gwen Knapp Center for Lupus and Immunology Research, University of Chicago, Chicago, IL 60637 USA

## Abstract

*Zbtb16*-encoded PLZF is a signature transcription factor (TF) that directs the acquisition of T-helper effector programs during the development of multiple innate lymphocyte lineages, including natural killer T cell, innate lymphoid cell, mucosal-associated invariant T cell and γδ lineages. PLZF is also essential in osteoblast and spermatogonial development. How *Zbtb16* itself is regulated in different lineages is incompletely understood. Here, by systematic CRISPR/Cas9-assisted deletions of chromatin accessible regions within the *Zbtb16* locus in mouse, we identify a critical enhancer controlling PLZF expression exclusively in innate lymphoid lineages. Multiple sites within this enhancer express canonical motifs for the TF Runx1, which is essential for the development of these lineages. Notably, some regulatory sites control the kinetic rather than the overall level of PLZF expression. Thus, our comprehensive, unbiased analysis of regulatory elements in vivo reveals critical mechanisms of *Zbtb16* regulation shared between innate and innate-like lymphoid lineages.

## Abstract (web summary)

*Zbtb16*-encoded transcription factor PLZF directs the differentiation of multiple innate and innate-like cell lineages, but how *Zbtb16* itself is regulated remains unclear. Here the authors show, using CRISPR gene editing, ATAC-seq and ChIP-seq, that specific Runx1-bound enhancer elements critically modulate lineage-dependent expressions of PLZF.

## Introduction

A hallmark of innate and innate-like lymphocytes is their acquisition of T-helper effector programs during development, independent of exogenous stimuli or exposure to infection^1–4^. In the bone marrow, a common innate lymphoid cell precursor (ILCP) gives rise to ILC1, ILC2 and ILC3 lineages expressing T-helper 1, Th2 and Th17 programs directed by T-bet, GATA3 and RORγt, respectively. Likewise, in the thymus, a common CD1d-restricted Vα14-Jα18 expressing thymic natural killer T cell (NKT) precursor gives rise to NKT1, NKT2 and NKT17 subsets. The acquisition of these innate helper effector programs depends on the specific induction of the signature transcription factor (TF) PLZF encoded by *Zbtb16* ^5, 6^, which directly binds and regulates key effector genes involved in lymphocyte migration, adhesion, costimulation, cytokine signaling as well as genes encoding master TFs controlling the T-helper programs^7^.

PLZF is not expressed in conventional lineages, whether resting or activated, indicating tight lineage-specific control of expression^8^. In the ILC lineage, PLZF is transiently expressed at the ILCP stage^9^, capping a partially defined sequence of TFs that are critically involved in early ILC development from a common lymphoid precursors (CLP) precursor, including *Nfil3*, *Tox*, *Tcf7* and *Id2*. How these TFs are induced in the first place and their potential interconnections remain poorly understood^10–12^. Furthermore, which of these or other TFs contribute to *Zbtb16* induction has not been defined.

In the NKT lineage, PLZF induction is dependent on Vα14-Jα18 TCR signaling elicited by recognition of CD1d-lipid ligands on thymocytes in the context of homotypic Slamf receptor engagement^1^. These signals result in elevated and sustained level of NFAT-induced Egr2, which directly activates the *Zbtb16* promoter^13, 14^. Other innate-like lymphoid lineages such as MR1-restricted Vα19-Jα33 mucosal-associated invariant T cell (MAIT) cells^5, 15^ and Vγ1/Vδ6 γδT cells^16^ also depend on the specific expression of PLZF during their development, likely through similar TCR-induced signals which remain to be studied.

Thus, PLZF appears to be broadly involved in the development of innate and innate-like T cells and their divergence from conventional lymphocytes. Furthermore, PLZF is also critically involved in some non-haematopoietic developmental processes, including limb patterning and spermatogonial self-renewal^17, 18^, raising intriguing issues regarding the mechanisms underlying this regulation of PLZF in multiple lineages. Cis-regulatory elements located in the non-coding regions of gene loci (termed enhancers) represent a major component of lineage-specific gene regulation^19^, but the potential function of enhancers in controlling PLZF expression has not been investigated.

To characterize the regulatory elements controlling PLZF expression during ILC and NKT precursor development, here we employ a systematic approach based on ATAC-seq^20^ to identify chromatin accessible regions in the *Zbtb16* locus of these lineages, and CRISPR/Cas9^21^ deletions to evaluate their individual impact on gene expression. By computational motif analysis, chromatin immunoprecipitation and conditional gene deletion, we identify the TFs bound to these regulatory elements. Our study reveals a shared lymphoid-specific enhancer essential for the induction of PLZF in both innate and innate-like lymphoid lineages and suggests a critical function of Runx1 in activating this enhancer.

## Results

### Chromatin accessibility at the *Zbtb16* locus

**Fig. 1:**
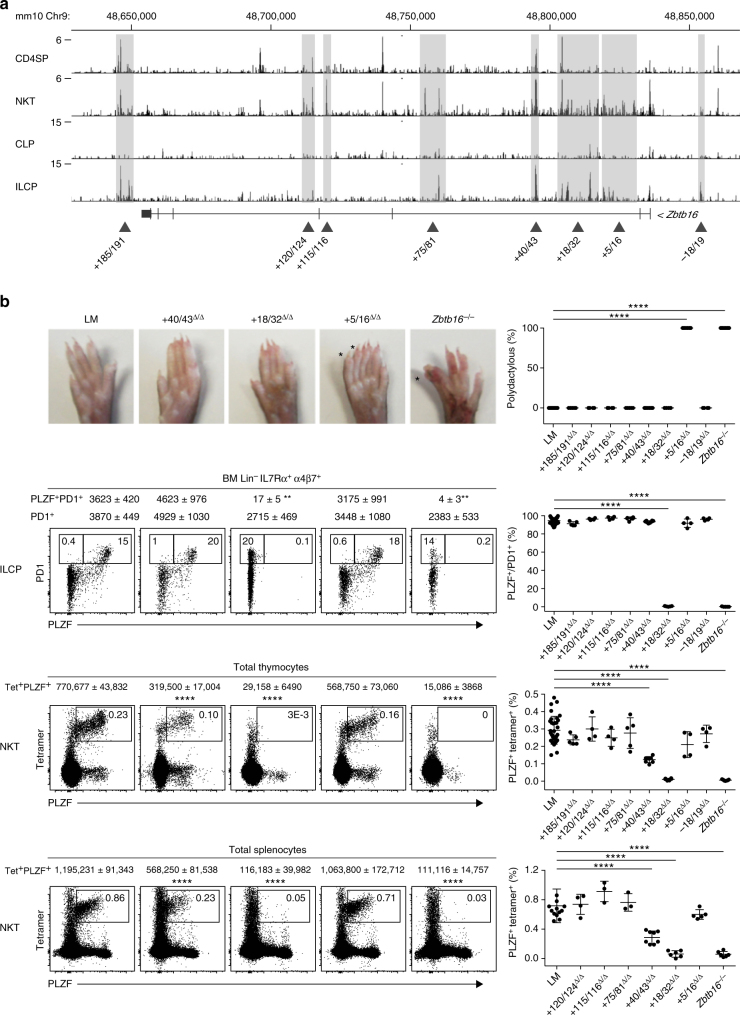
Global functional analysis of the *Zbtb16* locus by ATAC-seq and CRISPR/Cas9. **a** ATAC-seq profiles of the *Zbtb16* locus in CD4SP and NKT thymocytes, and in CLP and ILCP bone marrow cells. Shaded areas highlight clusters of peaks selected for CRISPR/Cas9 deletion based on differences in profiles between NKT vs CD4SP or ILCP vs CLP. Data are representative of two to three biological replicates. **b** *Top row* Hindlimb autopods of mice carrying different genomic deletions as indicated; extra digits or homeotic transformations of digits are labeled by an *asterix* and the frequency of mice with digit abnormalities is shown in the summary plot. *Second row* FACS analysis of PLZF and PD1 expression by BM Lin^-^IL7Rα^+^α4β7^+^ cells from mice carrying indicated CRISPR/Cas9 deletions; absolute numbers (mean ± S.E.M.) of ILCP (PD1^+^) and PLZF-expressing ILCP (PLZF^+^PD1^+^) are indicated on top of the FACS panels; the percentage of PLZF expression by ILCP is compiled in the summary scatter plot. *Bottom two rows* FACS analysis of CD1d-αGalCer tetramer and PLZF staining among total thymocytes and splenocytes of mice carrying indicated deletions; absolute numbers (mean ± S.E.M.) of NKT (CD1d-αGC Tetramer^+^PLZF^+^) and percentages are indicated on top of the FACS panels and in the summary scatter plots respectively. Summary data are pooled from 15 separate experiments, with a total of 4–42 mice in each group. Statistical analysis was performed using one-way ANOVA for multiple comparisons to WT littermate controls (LM). *P < 0.05, **P < 0.01, ***P < 0.001, ****P < 0.0001

To define regions of chromatin accessibility, we performed ATAC-seq analysis of freshly isolated NKT thymopcytes and bone marrow ILCP. Numerous ATAC-seq peaks were called by magnetic-activated cell sorting (MACS) software analysis along a ~200 kb segment encompassing the *Zbtb16* locus (Fig. 1a). The peaks were particularly abundant in intron 2 and often grouped in clusters. They were mostly present in thymic NKT or bone marrow ILCP, both of which express PLZF, and were less frequent in control CD4 single positive thymocytes (CD4SP) or CLP, which do not express PLZF.

### CRISPR/Cas9-mediated deletions of ATAC-seq peaks

For a first round of CRISPR/Cas9 deletions, the specific ATAC-seq peaks were grouped into eight transposase hypersentivity regions that together covered most of the individual ATAC peaks, as indicated in Fig. 1a. These regions ranged in size between 1 and 15 kb, and were designated by their position with respect to the transcription start site, including + 185/191, + 120/124, + 115/116, + 75/81, + 40/43, + 18/32, + 5/16 and −18/19. Each of these eight regions was deleted by the CRISPR/Cas9 method in B6 mouse zygotes microinjected with flanking pairs of single-guide RNAs. Mice carrying homozygous deletions were examined after at least five backcrosses to B6 mice if the deletion was associated with a developmental phenotype, and at least two backcrosses in the absence of apparent phenotype. To assess phenotypic alterations in NKT and ILC lineage development, we directly examined the level of expression of PLZF and associated changes in precursor frequencies by flow cytometry of thymus and bone marrow cells, respectively. For skeletal phenotypes, we scored the presence of hindlimb autopod and zygopod abnormalities, namely ectopic or homeotically transformed digits and fibulation of tibia, as described^18^. For each genomic region, homozygous deletion mutants (Δ/Δ) were compared with WT ( + / + ) littermates and with mice carrying a deletion of *Zbtb16* exon 2 (*Zbtb16* ^−/−^ mice).

Mice lacking segment + 5/16 exhibited morphological defects in hind limbs, including extra or homeotically transformed digits (Fig. 1b, top row) and fibulation of tibia (Supplementary Fig. 1), that are characteristic of previously described coding mutations of *Zbtb16* ^18^. In contrast, PLZF expression and cell frequencies were unaltered in bone marrow ILCP, identified by their Lin^-^ IL7Rα^+^ α4β7^+^ PD1^+^ phenotype, and in thymic and splenic NKT, identified by CD1d-αGalCer tetramer staining (Fig. 1b, bottom rows).

In mice carrying the adjacent + 18/32 deletion, the opposite phenotype was observed. These mutant mice lacked skeletal abnormalities, but had complete abrogation of PLZF expression, both in bone marrow ILCP and in thymic and splenic NKT (Fig. 1b). As a consequence, NKT thymocytes and splenocytes were massively decreased and ILCP were modestly decreased both in frequency and in absolute numbers, similar to *Zbtb16* ^−/−^ mice. Interestingly, expression of PLZF by γδ and MAIT thymocytes was also abolished (Supplementary Fig. 2). Another region, + 40/43, was associated with decreased NKT cell frequency, although the defect was less severe than + 18/32.

Mice carrying the other genomic deletions, including + 185/191, + 120/124, + 115/116, + 75/81 and −18/19 failed to exhibit lymphoid or skeletal developmental phenotypes (Fig. 1b).

Altogether, this systematic deletion analysis of the chromatin accessible regions specifically associated with bone marrow ILCP or NKT thymocytes indicates the presence of a limited number of regulatory regions controlling *Zbtb16* expression during the development of innate lymphoid lineages, and their clear separation from regions involved in the regulation of other developmental processes, such as limb patterning.

### NKT phenotypes in the + 18/32 and + 40/43 deletion mutants

**Fig. 2:**
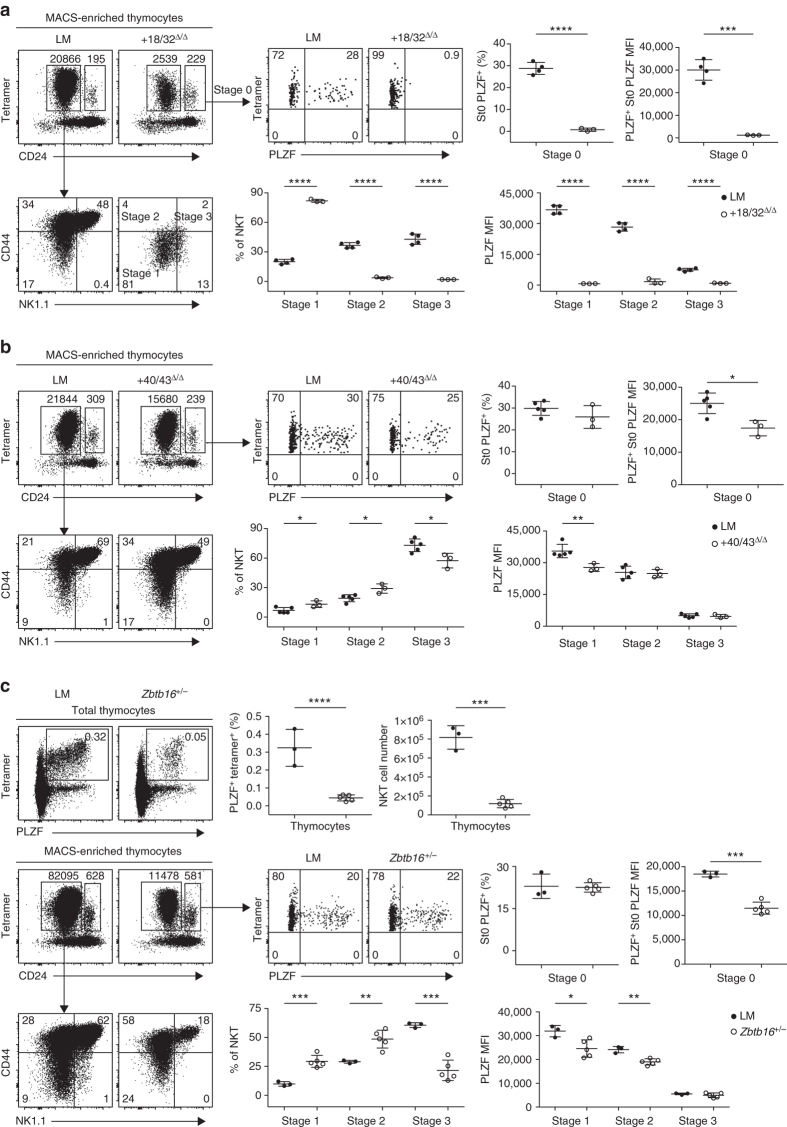
NKT phenotype of + 18/32 and + 41/43 deletions. **a** FACS analysis of CD1d-αGalCer-tetramer^+^ MACS-enriched NKT thymocytes in mice carrying the + 18/32 deletion compared with WT littermates (LM) mice. Stage 0 NKT (gated as Tetramer^+^ CD24^high^) are analyzed for PLZF expression (*upper panels*). Tetramer^+^ CD24^low^ NKT are stained for CD44 and NK1.1 to identify stages 1–3 and determine their percentages and PLZF mean fluorescence intensity (MFI) (*bottom row*). Summary data are combined from two independent experiments, with a total of three to four mice in each group. **b** FACS analysis of NKT developmental stages and PLZF levels in mice carrying the + 40/43 deletion compared with WT littermate (LM). Data are combined from two independent experiments, with a total of three to five mice in each group. **c** FACS analysis of NKT thymocytes in *Zbtb16* ^+/−^ mice. Two-tailed Student’s *t*-test was performed for statistical analysis. *P < 0.05, **P < 0.01, ***P < 0.001, ****P < 0.0001

Because PLZF levels vary considerably during different stages of normal NKT cell development^5, 6^, we performed a detailed examination of NKT thymocytes in mutant mice carrying the + 18/32 deletion, where the NKT cell defect is complete, and in the + 40/43 deleted mice, where the NKT cell defect is partial. After MACS-enrichment using CD1d-αGalCer tetramers, we could zoom in on the rare residual NKT thymocytes of + 18/32^Δ/Δ^ mice and show that they were arrested at the CD44^−^ NK1.1^−^ CD24^−^ stage 1 (Fig. 2a), similar to the previously described defect in *Zbtb16* ^−/−^ mice^5, 6^. We also examined the earliest CD44^−^ NK1.1^−^ CD24^hi^ developmental stage 0 cells^22^, which are normally present at very low numbers of around 300 cells per thymus and represent the stage where PLZF is first induced. Whereas, in WT littermates, ~30% of stage 0 cells expressed a high level of PLZF, expression was totally abrogated after deletion of + 18/32. Thus, the NKT developmental defect associated with the + 18/32 deletion is identical to the reported defect of *Zbtb16* ^−/−^ mice.

In + 40/43^Δ/Δ^ mice, a modest ( ~ 20–40%) but statistically significant defect in PLZF mean fluorescence intensity was observed in both stage 0 (*P* = 0.015; Student’s *t*-test) and stage 1 (*P* = 0.0084; Student’s *t*-test) cells, while later stages 2 and 3 NKT cells expressed similar levels of PLZF as wild type (Fig. 2b). This altered pattern of expression resulted in a partial block of NKT development, particularly visible at the stages 2 to 3 transition. These findings, which were confirmed in multiple independent experiments (Supplementary Fig. 3), indicate that NKT levels are exquisitely sensitive to modest changes in PLZF expression at early stages of development. This conclusion is in line with the similar NKT developmental defect observed in *Zbtb16* ^+/−^ heterozygous mice (Fig. 2c and Supplementary Fig. 3). In contrast with the NKT defect, the ILCP of + 40/43^Δ/Δ^ mice did not have alterations of PLZF expression (Fig. 1b), suggesting that + 40/43 specifically regulates NKT but not ILC development.

### Dissection of the + 18/32 region

**Fig. 3:**
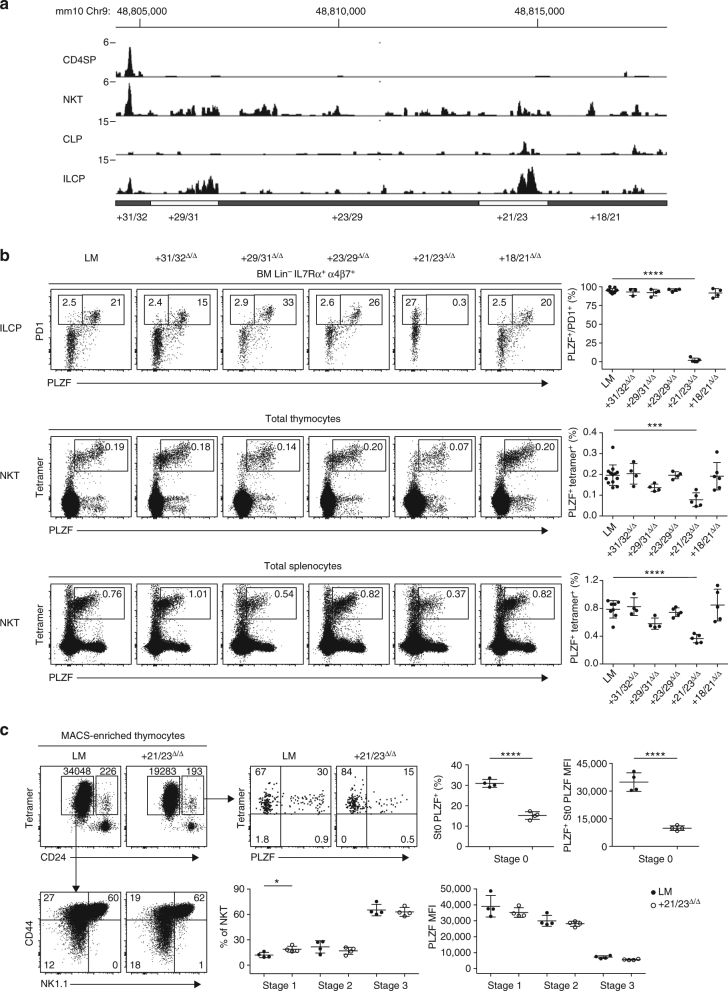
Dissection of the + 18/32 region. **a** ATAC-seq tracks. **b** FACS analysis of BM ILCP (*upper row*) and NKT thymocytes (*middle row*) and splenocytes (*bottom row*) in mice carrying the indicated deletions. Summary data in the right column are compiled from six independent experiments, with a total of 4–13 mice in each group. One-way analysis of variance for multiple comparisons was used for statistical analysis. **c** FACS analysis of NKT developmental stages and PLZF expression in + 21/23^Δ/Δ^ mice and littermate controls (LM). Data are combined from two independent experiments with a total of four mice per group. Two-tailed Student’s *t*-test was performed. *P < 0.05, **P < 0.01, ***P < 0.001, ****P < 0.0001

To further characterize the precise regulatory elements regulating ILCP and NKT development within the + 18/32 region, we generated five deletion mutants spanning this entire segment (Fig. 3a). Only one region, + 21/23, appeared to exert strong control on PLZF expression in ILCP and NKT cells (Fig. 3b). Indeed, mice carrying a deletion of + 21/23 exhibited undetectable PLZF expression in ILCP, similar to the larger + 18/32 deletion examined previously. In NKT thymocytes and splenocytes, the defect associated with the + 21/23 deletion was partial and did not fully recapitulate the + 18/32 deletion, suggesting the presence of additional ‘subthreshold’ regulatory elements that complement + 21/23. One such regulatory element may be encoded by + 29/31 whose deletion was associated with modestly decreased expression that did not reach statistical significance (Fig. 3b). Analysis of MACS-enriched NKT thymocytes lacking + 21/23 showed a transient but severe defect in PLZF expression at the early stage 0, which subsided at later stages. This defect involved both the fluorescence intensity and the frequency of PLZF expression among stage 0 cells, representing a delay in PLZF expression at stage 0, which appeared sufficient to significantly impair the frequency of total NKT thymocytes (*P* = 0.0002; ANOVA) and splenocytes (*P* < 0.0001; ANOVA) (Fig. 3b, c). Thus, again, transient defects in PLZF induction at early developmental stages, albeit with a slightly different kinetics compared with the + 40/43 mutants, were associated with substantial NKT developmental defects.

### Impact of PLZF defect on NKT sublineages

**Fig. 4:**
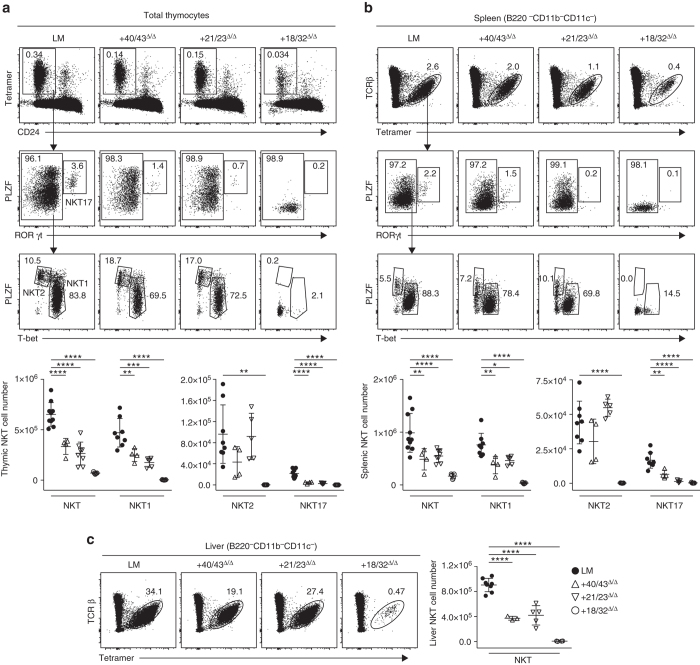
Impact on NKT sublineages. Intracellular FACS staining of PLZF, T-bet and RORγt in **a** thymic and **b** splenic NKT cells in indicated mutant mice, with summary panels. **c** Analysis of liver NKT cells. Data are from four independent experiments, with 4–10 mice in each group. Statistical analysis was performed using one-way ANOVA for multiple comparisons to WT LM. *P < 0.05, **P < 0.01, ***P < 0.001, ****P < 0.0001

The development of NKT thymocytes can also be described by their acquisition of T-helper types 1, 2 or 17 programs^1, 23, 24^, with NKT1, NKT2 and NKT17 characterized by their PLZF^low^ T-bet^high^, PLZF^high^ RORgt^low^ and PLZF^high^ RORgt^high^ profiles, respectively. We found that NKT1 and NKT17 were predominantly affected while NKT2 were relatively preserved in the thymus and peripheral tissues of + 21/23^Δ/Δ^ and in + 40/43^Δ/Δ^ mice, in which the decrease in PLZF expression is limited to the early developmental stages (Fig. 4 and Supplementary Fig. 4). In contrast, + 18/32^Δ/Δ^ mice lacked all NKT sublineages. Thus, early, transient defects in PLZF expression have long-range effects on terminally differentiated NKT sublineages.

### Sequence-level dissection of the + 21/23 region

**Fig. 5:**
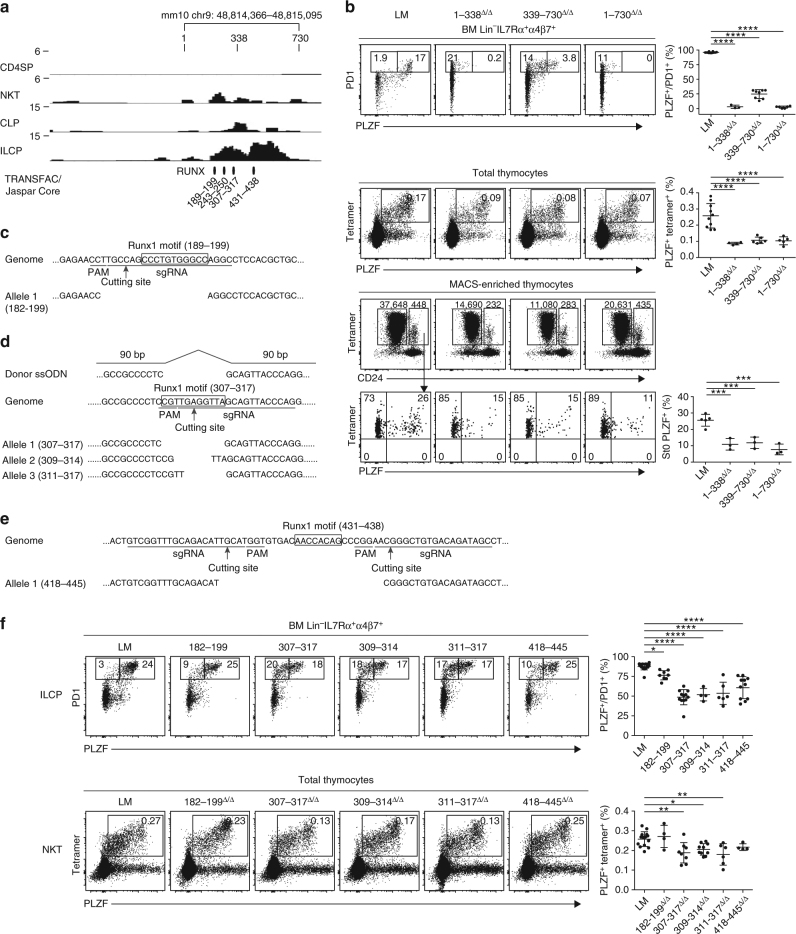
Sequence-level analysis of the + 21/23 region. **a** ATAC-seq profiles of the + 21/23 region in indicated lymphoid populations, magnified from Fig 1a, reveals that all reads are contained within a 730 bp located between mm10 chr9 48,814,346 and 48,815,095. **b** FACS analysis of BM ILCP (*top row*), and thymic NKT (*second row*) in mice carrying indicated deletions, with summary plots on the right column. Bottom two rows shows the FACS analysis of NKT stages 1-2-3 (CD24^low^) and NKT stage 0 (CD24^high^) after tetramer-MACS enrichment. The frequency of PLZF expression in stage 0 NKT cells are shown in representative FACS panels and in summary data pooled from two to three independent experiments, with 3–10 mice in each group. **c**–**e** Schematic representation of CRISPR/Cas9 targeting of different Runx1 motif-containing sequences. **f** FACS analysis of BM ILCP cells (*top row*), and thymic NKT cells (*bottom row*) in mice carrying indicated deletions, with summary plots on the right column. For ILCP analysis, Runx-motif mutant mice had the second allele carrying the + 18/32 deletion. Summary data are pooled from five independent experiments, with 4–17 mice in each group. One-way ANOVA with multiple comparisons compared to WT were used for statistical analysis. *, *P* < 0.05, **, *P* < 0.01, ***, *P* < 0.001, ****, *P* < 0.0001

Region + 21/23 contains ATAC-seq reads over a 730 base-pair interval (Fig. 5a). The precise deletion of this 730 bp fragment reproduced the phenotype of the larger + 21/23 deletion, as expected, with complete abrogation of PLZF expression in ILCP and impaired PLZF expression in stage 0 NKT thymocytes (Fig. 5b). We subdivided 1/730 with individual deletions of 1/338 and 339/730 segments. Both deletions reproduced the ILCP and NKT phenotypes of the larger 1/730 deletion, suggesting the presence of essential regulatory elements in both fragments. Since both fragments contained consensus DNA sequence motifs for Runx factor binding (Fig. 5a), we generated new deletions of three of these sequences. Excision of the entire 307/317 motif, as well as partial deletions of this motif (309/314 and 311/317) induced a significant decrease in PLZF expression by ILCP (*P* < 0.0001; analysis of variance (ANOVA)) as well as decreased NKT thymocytes (*P* = 0.0027; ANOVA) (Fig. 5d, f). Furthermore, a deletion centered around the 431/438 motif induced a significant decrease in PLZF expression in ILCP (*P* < 0.0001; ANOVA), and a modest decrease in NKT that did not reach significance (Fig. 5e, f). Mice lacking another Runx1 motif-containing sequence, 182–199 had modest but significant PLZF defect in ILCP (*P* = 0.026; ANOVA) (Fig. 5c, f). Together, these findings suggest that several Runx-motif-containing sequences within 1/730 contribute to regulation of *Zbtb16* in ILCP and in NKT, and therefore underlie the regulation of PLZF in both lineages.

### Runx1 binding at the *Zbtb16* locus

**Fig. 6:**
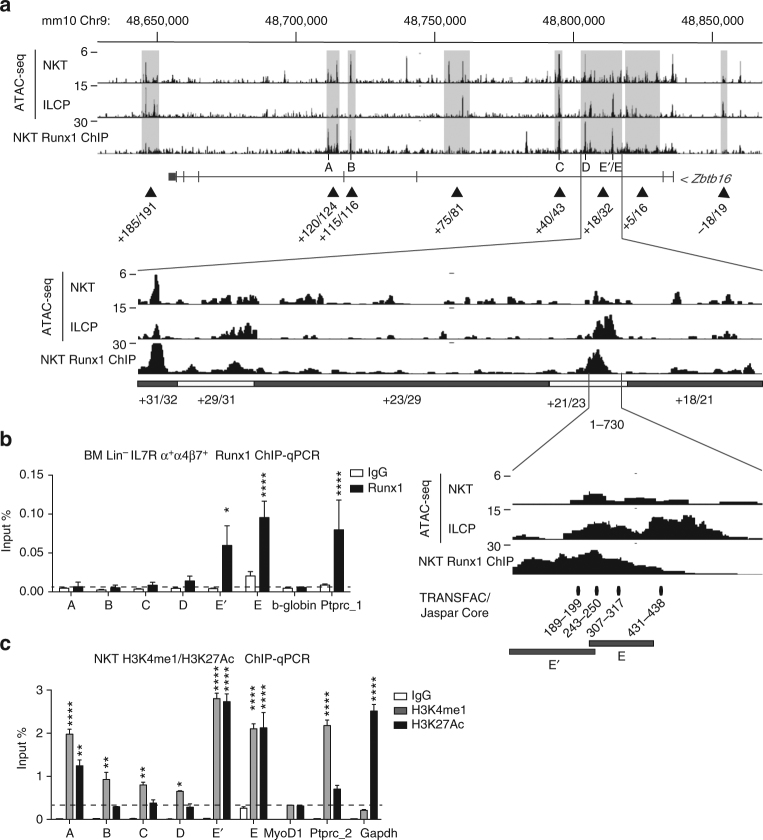
ChIP analysis of Runx1 binding. **a** Runx1 ChIP-seq profile of the *Zbtb16* locus in NKT thymocytes aligned with corresponding ATAC-seq profiles in NKT thymocytes and ILCP cells, with magnification of regions + 18/32 and 1/730 as indicated. **b** Runx1 ChIP-qPCR analysis of BM Lin^-^IL7Rα^+^α4β7^+^ cells at sites A–E as defined above. Ptprc-1 and b-globin were used as positive and negative control, respectively. Data are combined from three independent experiments. **c** H3K4me1 and H3K27Ac modifications at the *Zbtb16* locus. Ptprc-2 and Gapdh were used as positive controls for H3K4me1 and H3K27Ac, respectively. MyoD1 was used as negative control for both. Data are summarized from three independent experiments. Statistical analysis by one-way ANOVA for multiple comparisons. *P < 0.05, **P < 0.01, ***P < 0.001, ****P < 0.0001

By chromatin immunoprecipitation (ChIP)-seq analysis of NKT thymocytes with a Runx1-specific antibody (Supplementary Fig. 5), we demonstrated prominent binding of Runx1 to several regions of chromatin accessibility in the *Zbtb16* locus (Fig. 6a). Thus, Runx1 ChIP-seq peaks were found within the + 21/23, + 29/31, + 40/43, + 115/116 and + 120/124 regions in NKT thymocytes. Due to lower cell input, the ChIP analysis of ILCP was performed using ChIP-quantitative polymerase chain reaction (qPCR) and focused on the ILCP ATAC-seq regions. In this cell-type, Runx1 was found to bind exclusively to + 21/23, but not the other chromatin^−^accessible regions (Fig. 6b). Altogether, these results established that Runx1 bound heavily to the regulatory elements that control the expression of *Zbtb16* in NKT and in ILCP. The major involvement of the + 21/23 enhancer was consistent with the high density of H3K27Ac and H3K4me1 marks in this region (Fig. 6c). The findings also indicated that Runx1 bound to several other chromatin accessible regions that did not seem to be required for *Zbtb16* expression in NKT thymocytes, including + 185/191, + 120/124, + 115/116 and + 5/16, indicating that Runx1-binding at a particular site is not necessarily associated with active regulation of *Zbtb16* gene expression.

### PLZF expression in mice with conditional *Runx1* deletion

**Fig. 7:**
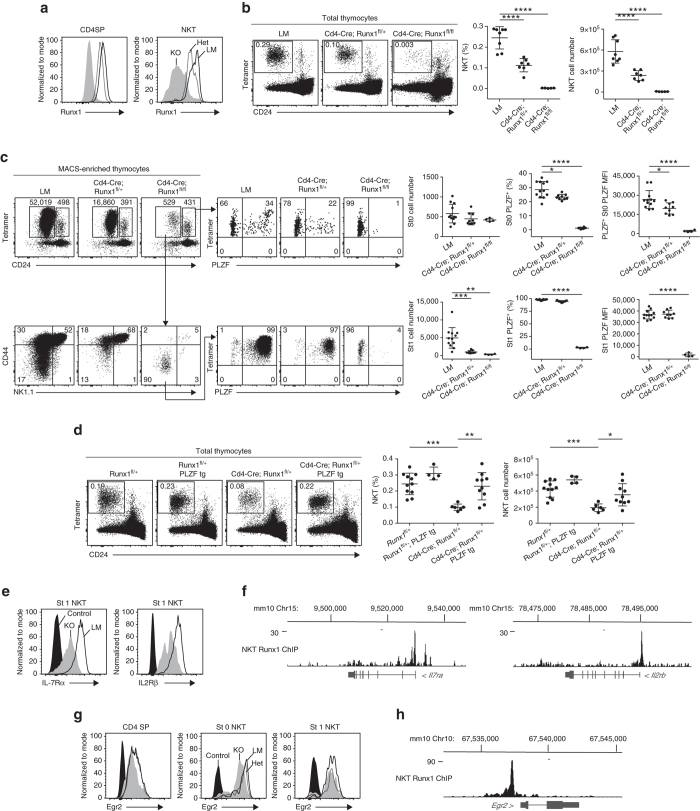
Conditional ablation of *Runx1* in NKT. **a** Intracellular flow cytometry for Runx1 expression in thymic CD4SP and NKT cells. (LM controls (LM), solid histogram; *Cd4*-*Cre Runx1* ^fl/+^ (Het), dashed histogram; *Cd4*-*Cre Runx1* ^fl/fl^ (KO), *gray shaded*.) **b** NKT cells in the thymus of Het and KO compared to their LM. The frequency and absolute cell number of NKT are summarized on the *right panels*. Data are representative of five to eight mice from four independent experiments. **c** Individual thymus of indicated strains were MACS-enriched using CD1d-αGalCer tetramers before FACS analysis of NKT developmental subsets and PLZF expression. Data are summarized from four independent experiments, with 4–12 mice in each group. **d** Rescue of NKT defect in Het by PLZF transgene expression. Data are representative of four to 11 mice from three independent experiments. **e** Representative FACS analysis of IL-7Rα and IL-2Rβ expression in stage 1 NKT thymocytes from LM and KO; mean ± S.E.M. of mean fluorescence intensity (MFI) is 5535 ± 331 (*n* = 4) vs 1501 ± 40 (*n* = 3) for IL-7Rα; and 5721 ± 87 (*n* = 4) vs 3040 ± 163 (*n* = 3) for IL-2Rβ, respectively. Leftmost histogram represents unstained control. **f** Runx1 ChIP-seq tracks at the *Il7ra* and *Il2rb* loci. **g** FACS analysis of Egr2 expression in CD4 SP, stage 0 and stage 1 NKT thymocytes from LM, Het and KO. Mean ± S.E.M. of Egr2 MFI: 2090 ± 84 (LM, *n* = 5), 1927 ± 45 (Het, *n* = 4) and 1506 ± 18 (KO, *n* = 6) in CD4 SP; 9688 ± 461 (LM, *n* = 5), 8872 ± 389 (Het, *n* = 4) and 5722 ± 316 (KO, *n* = 6) in stage 0 NKT; 4174 ± 287 (LM, *n* = 5), 4310 ± 257 (Het, *n* = 4), and 3892 ± 216 (KO, *n* = 6) in stage 1 NKT. **h** Runx1 ChIP-seq track at the *Egr2* locus. Statistical analysis was performed using one-way ANOVA with multiple comparisons. *P < 0.05, **P < 0.01, ***P < 0.001, ****P < 0.0001

To further evaluate the role of Runx1 on NKT cell development and PLZF expression in vivo, we used a *Cd4*-*Cre Runx1* ^fl/fl^ strain. As previously reported^25^, *Cd4*-*Cre Runx1* ^fl/fl^ mice exhibited a profound and selective defect in thymic NKT numbers compared with *Cd4*-*Cre* littermates, while CD4 and CD8 SP cells appeared normal (Fig. 7a, b). Furthermore, *Cd4*-*Cre Runx1* ^fl/+^ heterozygous mice also exhibited a defect, albeit less severe than homozygous littermates, demonstrating haploinsufficiency. After MACS-enrichment of rare residual NKT thymocytes using CD1d-αGalCer tetramers, we determined that the residual Runx1-deficient NKT thymocytes were arrested at stage 1 and that they failed to express detectable PLZF at stage 0 and stage 1 (Fig. 7c), a phenotype identical to that of + 18/32 deletion mutants shown in Fig. 2a and to that of *Zbtb16* ^−/−^ mice. Moreover, *Cd4*-*Cre Runx1* ^fl/+^ heterozygous mice exhibited defects in PLZF expression in stage 0 but not stage 1 NKT thymocytes, with a partial defect in total NKT thymocyte frequency (Fig. 7c). Thus, conditional deletion of *Runx1* alters PLZF expression in NKT precursors in a dose-dependent manner. Furthermore, crossing *Cd4-Zbtb16* transgenic mice to *Cd4*-*Cre Runx1* ^fl/+^ heterozygous mice partially but significantly rescued the defect in NKT percentage (*P* = 0.0025; Student’s *t*-test) and cell number (*P* = 0.017; Student’s *t*-test) (Fig. 7d), supporting the conclusion that Runx1 promotes NKT cell development in part by upregulating PLZF expression. In addition, Runx1 bound and partially regulated the expression of *Il7ra*, *Il2rb* and *Egr2*, suggesting other levels of regulation of NKT development (Fig. 7e–h), perhaps accounting for the incomplete rescue of Runx1-deficient NKT development by the PLZF transgene. Although Egr2 could directly regulate PLZF^13^, its modest downregulation in Runx1-deficient NKT thymocytes was insufficient to account for the complete loss of PLZF.

**Fig. 8:**
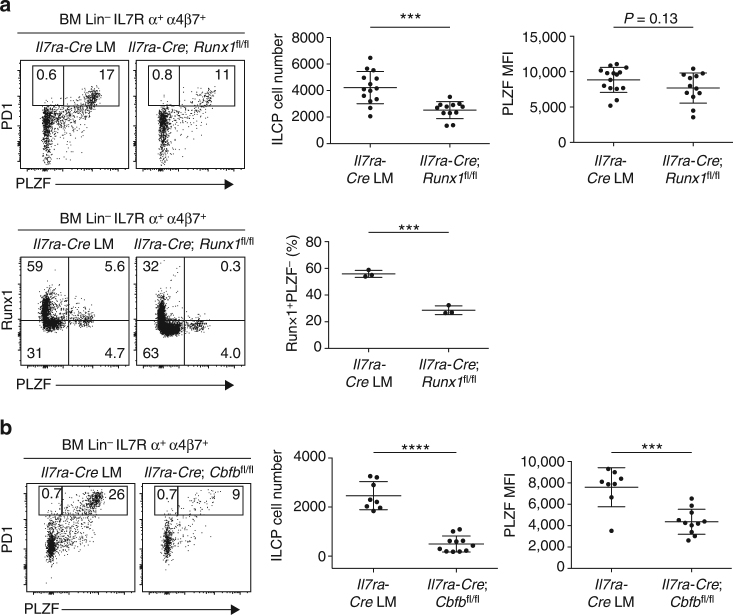
Conditional ablation of *Runx1* in ILCP. **a** *Top* PLZF expression in BM Lin^-^IL7Rα^+^ α4β7^+^ cells from *Il7ra*-*Cre* and *Il7ra*-*Cre Runx1* ^fl/fl^. BM samples were MACS-enriched using anti-α4β7 antibody before staining with other antibodies. Summary data of ILCP cell number and PLZF mean fluorescence intensity of ILCP cells are combined from seven independent experiments with 15 and 12 mice in each group. Bottom, Runx1 and PLZF staining in BM Lin^-^IL7Rα^+^ α4β7^+^ cells from *Il7ra*-*Cre* and *Il7ra*-*Cre Runx1* ^fl/fl^. Data are representative of three mice from each group. **b** PLZF expression in BM Lin^-^IL7Rα^+^ α4β7^+^ cells from *Il7ra*-*Cre* and *Il7ra*-*Cre Cbfb* ^fl/fl^. Data are summarized from four independent experiments, with 8–11 mice in each group. Statistical analysis was performed using two-tailed Student’s *t*-test. *P < 0.05, **P < 0.01, ***P < 0.001, ****P < 0.0001

To evaluate the role of *Runx1* on ILCP development, we used an *Il7ra*-*Cre Runx1* ^fl/fl^ strain to direct deletion of *Runx1* after the CLP stage. We observed modest but significant decrease in ILCP cell number (*P* = 0.0002; Student’s *t*-test) and a trend toward decreased PLZF expression compared with *Il7ra*-*Cre* controls, consistent with a role of *Runx1* in ILCP development (Fig. 8a). There was only partial decrease of Runx1 among BM Lin^−^ IL7Rα^+^ α4β7^+^ ILC precursors, however, perhaps due to a delayed kinetics of *Il7ra*-*Cre*-mediated excision (Fig. 8a, bottom). In addition, the known redundancy of Runx1 with other Runx proteins, particularly Runx3 which is expressed at the ILCP stage, might explain the modest phenotype. We therefore examined *Il7ra*-*Cre Cbfb* ^fl/fl^ mice lacking the common partner to all Runx proteins. Fig. 8b demonstrated a statistically significant defect in both ILCP numbers (*P* < 0.0001; Student’s *t*-test) and in their expression of PLZF (*P* = 0.0002; Student’s *t*-test). Thus, in contrast with NKT where Runx1 and Runx3 have little overlap (Supplementary Fig. 6) and deletion of Runx1 alone is associated with a complete developmental block, Runx3 might partially compensate for the loss of Runx1 in ILCP.

Altogether, these results demonstrated the critical role of Runx1 and the shared intronic enhancer bound by Runx1 in regulating PLZF expression during ILC and NKT development.

## Discussion

The TF PLZF is tightly regulated in a lineage-specific as well as temporal-specific manner to direct diverse lymphoid, skeletal and spermatogonial development programs, a pattern highly suggestive of enhancer-mediated regulation. By combining ATAC-seq and CRISPR/Cas9 analysis, our study identified a critical regulatory element within the intronic region + 21/23 that was critical for PLZF expression in both NKT and ILC lineages, as well as in MAIT and γδ cells. This region carried a high density of H3K27Ac and H3K4me1 marks and was heavily bound by Runx1. Although our study did not provide formal proof that Runx1 binding directly activated the enhancer, this region contained several Runx motifs whose individual deletions impaired PLZF expression in both ILC and NKT precursors. Furthermore, conditional deletion of *Runx1* led to defective PLZF expression and impaired lineage development, which was partially rescued by transgenic expression of PLZF. Runx1 also exerted modest regulation of other factors involved in development, such as IL7Rα, IL2Rβ and Egr2, which might explain why the Runx1 deficiency phenotype was only partially rescued by transgenic PLZF. Altogether, these findings demonstrate that Runx1 is a critical TF and suggest that it directly activates PLZF at a key enhancer shared by ILC and NKT lineages. They reveal the existence of a Runx1-PLZF axis as a central component of the transcriptional network underlying the development of innate lymphoid lineages.

Existing studies suggested that in mouse and human innate lymphoid lineages, *Zbtb16* contains extended ‘super-enhancer’ regions^26, 27^ that are characterized by diffuse H3K27 acetylation marks, a hallmark of highly transcribed lineage ‘identity’ genes. Indeed, we found numerous chromatin accessible regions in ILCP and NKT which were generally absent from related populations lacking PLZF expression, such as CLP and CD4 SP. It is notable in that regard that, despite the apparent complexity of the chromatin accessible landscape of *Zbtb16*, only a few chromatin accessible regions demonstrated functional relevance upon CRISPR/Cas9 deletion, indicating that many of them did not carry essential function in regulating *Zbtb16*, at least during the developmental period. Thus, by performing first a limited number of deletions involving large clusters of ATAC-seq peaks to cover the extended gene locus, it was possible to rapidly zoom, in an unbiased manner and with great specificity on a key regulatory element of a few hundred base pairs. Motif analysis within this enhancer, combined with transcriptional profiling led to the characterization of the central role of Runx1 in PLZF regulation and innate lymphoid development. Notably, Runx1 was also bound to several other chromatin accessible regions within the *Zbtb16* locus that did not appear to impact PLZF expression or NKT development, as well as to the promoter region, emphasizing the importance of site-specific deletions to identify the relevant elements.

Runx1 is involved at multiple but discrete stages of haematopoiesis and lymphopoiesis, including the development of B and T lymphoid progenitors, the lymphoid tissue inducer, as well as the differentiation of regulatory T cells and Th17 cells^28–30^. Furthermore, Runx1 was previously reported to be essential for NKT development^25^, although the stage and the mechanism of its action were not defined. Interestingly, multiple Runx1-binding motifs located within the same enhancer were implicated in PLZF regulation, with individual sequences contributing partial effects, similar to other well-studied models of Runx-mediated gene regulation, for example in *Cd4* gene repression^31^. In fact, deletions of individual Runx1 motifs revealed partial phenotypes related to the unexpected impact of relatively modest alterations of the level or the kinetics of early PLZF expression on the stage progression and the final frequency of NKT cells. Thus, a transient defect limited to the very early stage of NKT cell development was associated with long-range impact in the development of terminally differentiated NKT sublineages, particularly NKT1 and NKT17. Examples of these alterations were found in + 21/23^Δ/Δ^, + 41/43^Δ/Δ^ and *Cd4*-*Cre Runx1* ^fl/+^ mice, as well in *Zbtb16* ^+/−^ mice. As broad variations in NKT cell frequencies have been reported between different mouse strains and in humans, our findings suggest that relatively minor alterations of PLZF expression at early thymic stages, which would go undetected if not specifically investigated, could account for significant variations in NKT development.

Our study also identifies an adjacent but distinct skeletal specific enhancer region controlling limb patterning. The non-overlapping pattern of enhancers governing PLZF expression in lymphoid and in osteoblast precursors is consistent with emerging knowledge about gene regulation in different mammalian tissues. Furthermore, as osteoblast differentiation is characteristically triggered by Runx2 expression in mesenchymal cells^32^ before expression of PLZF^18^, it is intriguing to speculate that an analogous Runx2-PLZF axis may govern bone formation as well.

While traditional approaches to dissect the regulation of gene expression begin with a candidate TF and search for potential target sites, our study demonstrates the value of combining genome wide methods such as ATAC-seq and CRISPR/Cas9 to identify the genomic regulatory element first and then search for candidate TFs based on motif analysis and transcriptional context. This combination of approaches has the advantage of being systematic and unbiased, and is readily applicable to a wide range of systems. In addition, more refined epigenetic studies to identify poised or active enhancers, which were not available in our study due to low cell input, can potentially narrow down the number of targets for CRISPR/Cas9 analysis. While this approach is extremely powerful to identify key enhancers and focus the investigations on well-defined regulatory elements, there remain limitations in dissecting the full complement of factors regulating the enhancers, for example in the absence of obvious consensus binding motif. In that regard, it is possible that factors other than Runx1 may also regulate the common NKT/ILC enhancer.

Another advantage of this ATAC-seq/CRISPR/Cas9 combination approach is that valuable enhancer mutant model strains can be generated with selective defects of some but not other PLZF functions. In the current study, for example, + 21/23^Δ/Δ^ mice exhibited PLZF defects across the innate lymphoid system, without associated defects in tissues such as bone and sperm which have confounded or complicated the studies of PLZF-deficient mice.

In summary, we report the successful combination of ATAC-seq and CRISPR/Cas9 methods to systematically dissect the extended *Zbtb16* locus in vivo, revealing a shared lymphoid enhancer and the critical role of Runx1 in PLZF expression and the development of innate and innate-like lineages. These findings further advance our understanding of the architecture of the common transcriptional regulatory network that governs a fundamental lineage decision between innate and adaptive lymphocyte development.

## Methods

### Mice

C57BL/6J (stock no. 000664), B6.129P2-*Runx1* ^*tm1Tani*^/J (stock no. 008772), *Cbfb* ^*tm2.1Ddg*^/J (stock no. 028550) and *Cd4*-*Cre* (B6.Cg-Tg(CD4-cre)1Cwi/J (stock no 017336) mice were obtained from The Jackson Laboratory. *Zbtb16* ^−/−^ mice were a gift from Dr. P.P. Pandolfi (Beth Israel Deaconess Medical Center, Boston, MA) and were backcrossed to C57BL/6J for at least nine generations^18^. The *Zbtb16*-*IRESGFPcre* (B6(SJL)-*Zbtb16* ^*tm1.1(EGFP/cre)Aben*^/J) (stock no. 024529) and the C57BL/6J *Cd4*-*Zbtb16* transgenic (stock no. 014644) mice were generated^9, 33^ and maintained in our laboratory. C57BL/6J *Il7Ra*-*Cre* was obtained from Dr Rodewald^34^. All the CRISPR/Cas9-mediated knockout mice were generated by microinjection of C57BL/6 zygotes in the transgenic core service of the University of Chicago. The age of mice was between 4- and 8-week old for all the experiments performed. All mice were raised in a specific pathogen-free environment, and killed by CO_2_. The permission was granted to perform all mice experiments by the Institutional Animal Care and Use Committee of the University of Chicago.

### Flow cytometry and MACS enrichment

Bone marrow was isolated by gently crushing femurs and tibias before filtrations with 70 μm filters. Cell suspensions were incubated with purified anti-CD16/32 (clone 93) for 10 min on ice to block Fc receptors. The following fluorochrome-labeled monoclonal antibodies were used: α4β7 (DATK32; 1:200), B220 (RA3-6B2; 1:400), CD3ε (17A2; 1:400), CD4 (L3T4; 1:400), CD8α (53-6.7; 1:400), CD11b (M1/70; 1:400), CD11c (N418; 1:400), CD19 (6D5; 1:400), CD25 (PC61; 1:200), CD45.2 (104; 1:100), cKit (2B8; 1:100), Flt3 (A2F10; 1:100), Gr-1 (RB6-8C5; 1:400), IL2Rβ/CD122 (5H4; 1:50), IL-7Rα/CD127 (A7R34; 1:20), NK1.1 (PK136; 1:200), PD1 (J43; 1:50), Sca-1 (D7; 1:500), TCRβ (H57-597; 1:400) and Ter-119 (TER-119; 1:400). All the antibodies were purchased from eBioscience, BD Biosciences or BioLegend. For intracellular staining, cells were fixed and permeabilized using the eBioscience Foxp3 Staining Buffer Set and then stained with fluorochrome-labeled Egr2 (12-6691-82; eBioscience; 1:50), PLZF (563490 or 564850; BD Biosciences; 1:200 or 1:1000), RORγt (564723; BD Biosciences; 1:200), Runx1 (12-9816-80; eBioscience; 1:100), Runx3 (ab135248; Abcam; 1:500) and T-bet (644810; Biolegend; 1:2000) antibodies. For pre-enrichment of BM ILCP cells, samples were stained with APC-conjugated anti-α4β7 antibody, bound to anti-APC microbeads (Miltenyi Biotec), and then subjected to positive selection on autoMACS (Miltenyi Biotec). ILCP were sorted as Lin^−^IL-7Rα^+^α4b7^+^eGFP^+^. For isolation of CLP, lineage^+^ cells were depleted by staining with biotin-conjugated antibodies against B220, CD3ε, CD4, CD8α, CD11b, CD11c, CD19, Gr-1, NK1.1, TCRβ and Ter119, followed by incubation with streptavidin (SAV) microbeads (Miltenyi Biotec). CLP were sorted as Lin^−^IL^−^7Rα^+^cKit^int^Sca-1^int^Flt3^high^. CD1d-αGalCer and MR1-5-OP-RU tetramers were obtained from the NIH tetramer facility. For NKT or MAIT cell MACS enrichment, samples were labeled with APC-conjugated CD1d-αGalCer (1:400) or MR1-5-OP-RU (1:1000) tetramers, respectively, bound to anti-APC or anti-PE magnetic beads, and run on an autoMACS cell separator. Samples were analyzed on an LSRII (BD Biosciences) or sorted on fluorescence-activated cell sorting (FACS) Aria II (BD Biosciences), with doublet exclusion and DAPI staining of dead cells in most experiments. Data was analyzed by FlowJo (Tree Star).

### ATAC-seq

ATAC-seq was performed as described^20^. Nuclei were prepared from sorted CD4SP, NKT, CLP and ILCP cells (10,000–50,000 for each ATAC-seq) and resuspended in the transposase reaction mix (FC-121-1030; Illumina). The transposition reaction was carried out at 37 °C water bath for 30 min. Then the samples were purified using a Qiagen PCR MinElute kit (28006; Qiagen). Following purification, library fragments were amplified using Nextera PCR Primers (FC-121-1011; Illumina) and NEBnext PCR master mix (0541; New England Lab) for a total of 10–12 cycles. The libraries were then purified using a Qiagen PCR MinElute kit and size selected in the 150–650 bp range. The size-selected libraries were quantified using the Agilent Bioanalyzer and by qPCR using the KAPA Library Quantification Kit. Libraries were sequenced on the Illumina Hiseq2000 system to generate 30–50 million reads. ATAC-seq raw reads of 50 bp were aligned to the mm10 mouse genome using bowtie allowing for two mismatches. The bedgraphs of ChIP-seq were generated using hypergeometric optimization of motif enrichment (HOMER), where the total number of aligned reads was normalized to 10 million. For each cell type, two or three biological replicates were performed.

### Microinjection into mouse zygotes

Vectors pT3TS-nCas9n (46757) and pT7-gRNA (46759) were obtained from Addgene. A pair of oligos for each targeting site (Supplementary Table 1) was annealed and ligated to linearized pT7-gRNA vector. Linearized pT3TS-nCas9n was purified and used as the template for in vitro transcription (IVT) using mMESSAGE mMACHINE T3 Transcription Kit (AM1348; ThermoFisher Scientific). The ligated sgRNA constructs were linearized, purified and then used as the template for IVT using MEGAshortscript T7 Transcription Kit (AM1354; ThermoFisher Scientific). Both the Cas9 mRNA and the sgRNAs were purified using MEGAclear kit (AM1908; ThermoFisher Scientific) and eluted in RNase-free water.

C57BL/6J females at 9–15 weeks of age were screened for non-estrus state and were superovulated with 5-5.5 IU PMSG (Millipore). 46.5 h later mice were given 5-5.5 IU HCG (Sigma) and mated 1.5 h later to C57BL/6J males. The next morning the females were checked for the presence of a vaginal plug, and those having one were harvested for fertilized eggs. Injection of the CRISPR reagents into the pronucleus was performed by using an Eppendorf FemptoJet. Surviving eggs were transplanted in CD-1 pseudopregnant females. The founders were screened with PCR followed by sequencing. The sgRNAs and corresponding deletions obtained are listed in Supplementary Table 1.

### ChIP-seq and ChIP-qPCR

For Runx1 ChIP-seq in NKT cells, 20 million MACS-enriched NKT cells from Vα14-Jα18 tg mice were fixed for 15 min at room temperature with 10% formaldehyde. Glycine was then added to a final concentration of 0.125 M to quench the reaction. Cells were washed twice with ice-cold phosphate-buffered saline, lysed and followed by sonication to fragment DNA to 100–200 bp. The chromatin was then incubated overnight with Runx1 antibody (ab23980; Abcam), followed by 2 h incubation with Protein G Dynabeads (10003D; Invitrogen). Precipitated ChIP and input DNA were washed, reverse crosslinked and digested with proteinase K and RNase A. The DNA was then purified with phenol/chloroform exaction or Qiagen PCR MinElute kit. Sequencing libraries were prepared by standard end-repair, adenylation, adaptor-ligation, PCR amplification and size-selection procedures. For Runx1 ChIP-qPCR in BM Lin^−^ IL7Rα^+^ α4β7^+^ cells, BM cells were MACS-enriched with anti-α4β7 before staining with other antibodies and sorting. About 25,000–50,000 cells from 10 to 20 mice were used for each biological replicate. The primers used for ChIP-qPCR are listed in Supplementary Table 2.

### Immunoprecipitation and western blot analysis

293 cells (CRL-1573; ATCC) were transfected with Flag-mRunx1 construct (14585; Addgene). Cells were lysed with RIPA buffer 24 h post transfection and immunoprecipitated with IgG control, Runx1 (ab23980; Abcam) or Flag (F7425; Sigma) antibodies. Western blot was performed with anti-Runx1 (ab23980; Abcam) and anti-Flag-HRP (A8592; Sigma).

### Statistical analysis

Two-tailed Student’s *t*-test or one-way ANOVA with multiple comparisons to control were performed with Prism (GraphPad Software).

### Data availability

The ATAC-seq and ChIP-seq data have been deposited in NCBI GEO under the accession code GSE98662.

## Electronic supplementary material

Supplementary Information

Peer Review File
