# Publisher Correction: A shared Runx1-bound *Zbtb16* enhancer directs innate and innate-like lymphoid lineage development

**DOI:** 10.1038/s41467-017-01780-1

**Published:** 2017-11-30

**Authors:** Ai-Ping Mao, Isabel E. Ishizuka, Darshan N. Kasal, Malay Mandal, Albert Bendelac

**Affiliations:** 1 Committee on Immunology, University of Chicago, Chicago, IL 60637 USA; 2 Department of Pathology, University of Chicago, Chicago, IL 60637 USA; 3 Department of Medicine, Section of Rheumatology and Gwen Knapp Center for Lupus and Immunology Research, University of Chicago, Chicago, IL 60637 USA

*Nature Communications* **8**:863 doi:10.1038/s41467-017-00882-0; Article published online 16 October 2017

In the original PDF version of this Article, which was published on 16 October 2017, the publication date was incorrectly given as 11 October 2017. This has now been corrected in the PDF; the HTML version of the paper was correct from the time of publication.

