# Supplementary material for: A shared Runx1-bound Zbtb16 enhancer directs innate and innate-like lymphoid lineage development
Source: Nat Commun. 2017 Oct 16;8:863. doi: 10.1038/s41467-017-00882-0 (PMC5643357; doi:10.1038/s41467-017-00882-0)
Supplement: Supplementary file 1 — Supplementary Information [file 41467_2017_882_MOESM1_ESM.pdf]

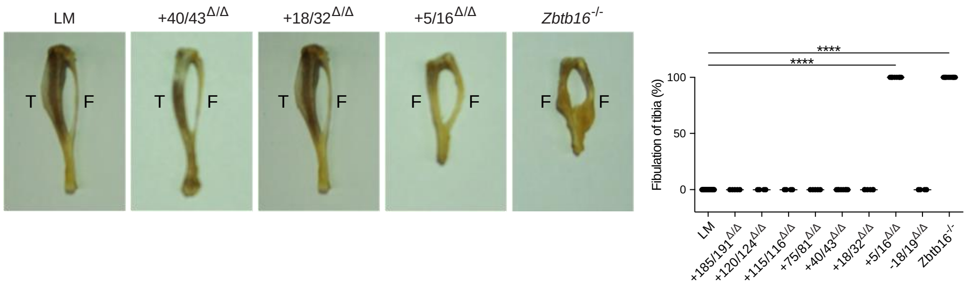

Supplementary Figure 1. Zeugopod abnormalities in deletion mutant mice. Left, the fibulation of tibia observed in +5/16$^{\Delta/\Delta}$ deletion mutant mice is identical to the PLZF$^{-/-}$ luxoid defect. Right, summary plot of zeugopod abnormalities among deletion mutant mice. Summary data are pooled from 15 separate experiments, with a total of four to 42 mice in each group. Statistical analysis was performed using one-way ANOVA for multiple comparisons to WT littermate controls (LM). *, $P < 0.05$, **, $P < 0.01$, ***, $P < 0.001$, ****, $P < 0.0001$.

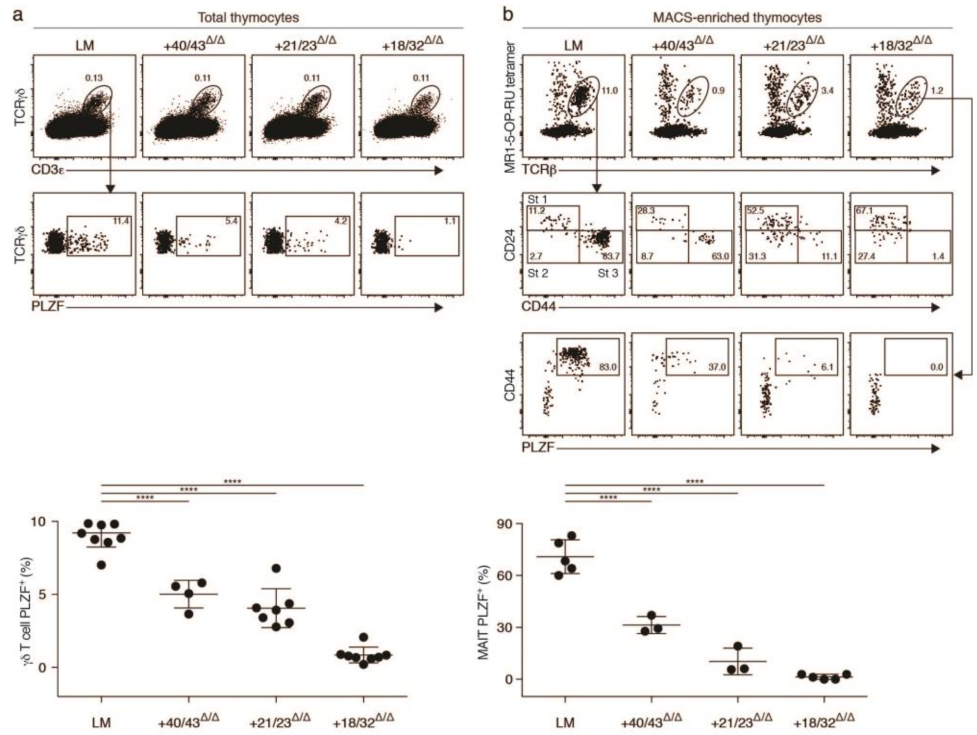

Supplementary Figure 2. PLZF expression in γδT and MAIT thymocytes of +40/43$^{\Delta/\Delta}$, +21/23$^{\Delta/\Delta}$, +18/32$^{\Delta/\Delta}$ mice. (**a**) thymic γδT cells. (**b**) thymic MAIT cells after MACS-enrichment with MR1-5-OP-RU tetramers. Data were compiled from four independent experiments with three to eight mice in each group. Statistical analysis was performed using one-way ANOVA for multiple comparisons. *, $P < 0.05$, **, $P < 0.01$, ***, $P < 0.001$, ****, $P < 0.0001$.

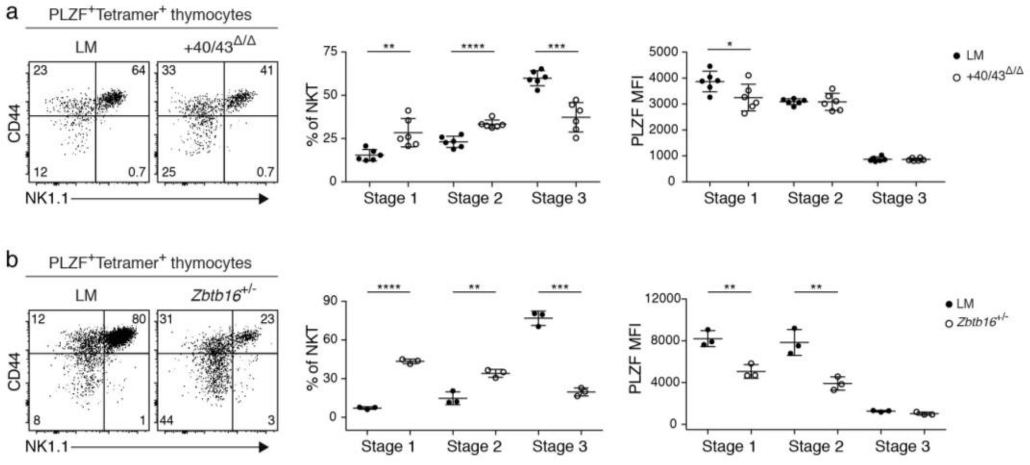

Supplementary Figure 3. **NKT cell defects in +40/43$^{\Delta/\Delta}$ and PLZF$^{+/-}$ mice**. Additional batches of mice were examined in independent experiments to confirm results shown in **Fig. 2b** and **Fig. 2c**. Summary data are pooled from two separate experiments, with a total of three to six mice in each group. Two-tailed Student's t test was performed for statistical analysis. *, $P < 0.05$, **, $P < 0.01$, ***, $P < 0.001$, ****, $P < 0.0001$.

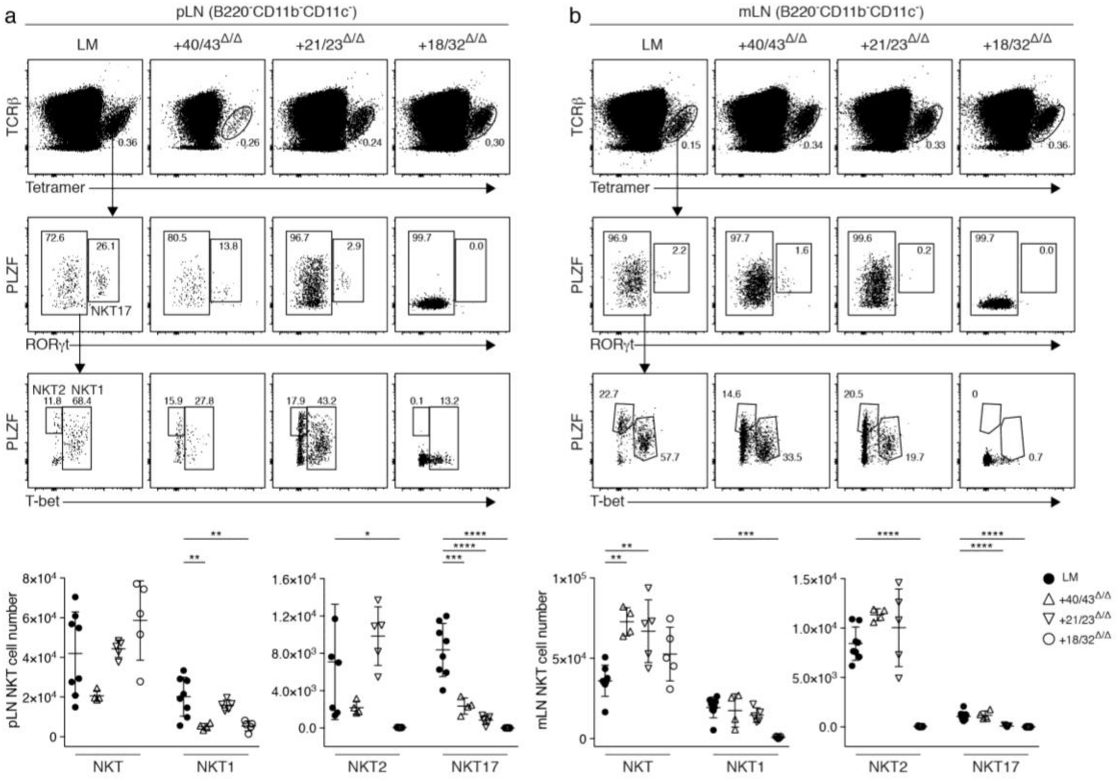

Supplementary Figure 4. NKT sublineages in lymph nodes of mutant mice. FACS analysis of TF expression by NKT cells from peripheral axillary and inguinal lymph nodes (pLN) (**a**) and mesentric lymph nodes (mLN) (**b**) of mice carrying the indicated enhancer deletions**.** Summary plots combining four independent experiments, with four to eight mice in each group. Statistical analysis was performed using one-way ANOVA for multiple comparisons to WT LM. *, $P < 0.05$, **, $P < 0.01$, ***, $P < 0.001$, ****, $P < 0.0001$.

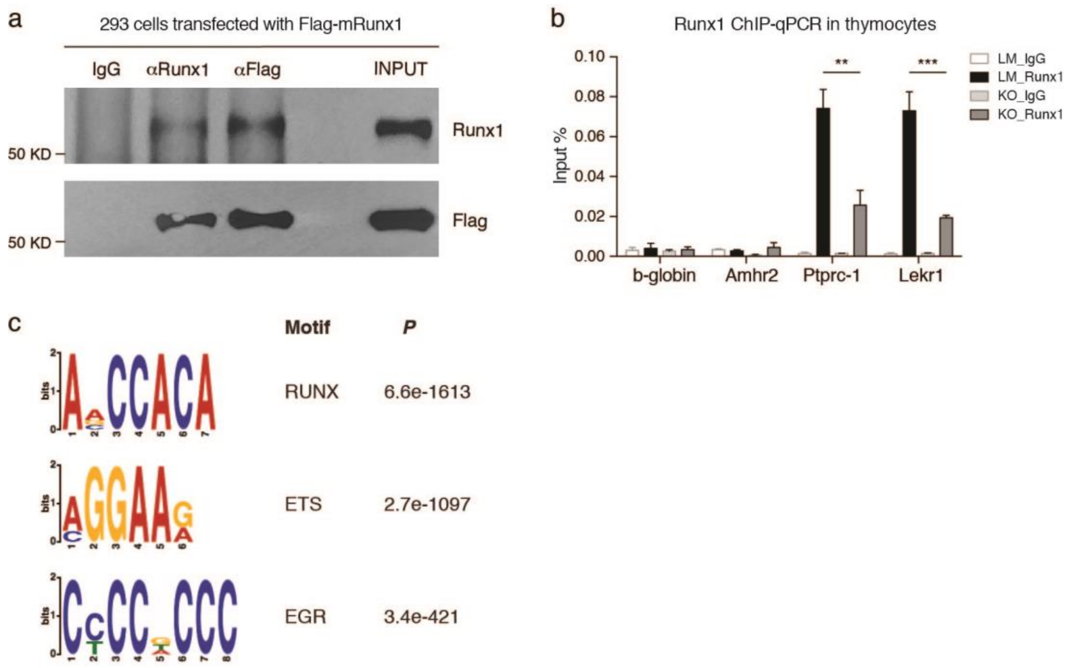

Supplementary Figure 5. Specificity of ab23980 anti-Runx1 antibody from Abcam. (a) IP/WB analysis of 293 cells transfected with mouse Flag-Runx1 construct. (b) Runx1 ChIP-qPCR of LM or Cd4-Cre Runx1fl/fl whole thymocytes. Note that the *Cd4-Cre Runx1*$^{fl/fl}$ thymocytes have some residual Runx1 signal from DN thymocytes which express high Runx1. Two-tailed Student's t test was performed for statistical analysis. *, *P* < 0.05, **, *P* < 0.01, ***, *P* < 0.001, ****, *P* < 0.0001. (c) The top three motifs identified by MEME-ChIP for Runx1 ChIP-seq peaks. We extracted 200 bp centered on all Runx1 ChIP-seq peaks and used as input for MEME-ChIP, set to use the Vertebrate (In vivo and in silico) motifs. The *P* value of Fisher's Exact Test for enrichment of the motif in the positive sequences is used.

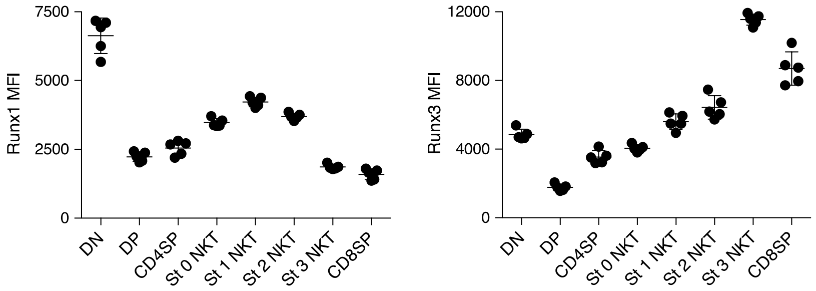

Supplementary Figure 6. Opposite expression patterns of Runx1 and Runx3 in NKT thymic stages. Flow cytometry analysis of intracellular Runx1 (left) and Runx3 (right). DN, DP, CD4SP and CD8SP are shown for reference. Five mice from two independent experiments were used for both Runx1 and Runx3 staining.

Supplementary Table 1. sgRNA and corresponding deletions.

Region	sgRNA	mm10 Chr9 sequence deletion
-18/19	GGCTATGCACACAAGCTCGG	48,853,652-48,854,902
	GGTAAATAGAAGCGGGGTAG	
+5/16	GGGGATAGGCACAAGAGCTC	48,819,509-48,830,792
	GGACGCGGGGCGAAAATGAT	
+18/32	GGTACCTCTTCACATGTGTA	48,804,391-48,818,263
	GGAGGGTTAGCTTAGACTTT	
+40/43	GGCGACCACATGTTACAGCC	48,793,359-48,795,508
+75/81	GGTAATGAACTAGAACGCCA	48,754,891-48,761,059
	GGTTGAGCTTTCTGAGGGGT	
+115/116	GGCTAATGATTCAGGATAAG	48,719,978-48,720,522
	GGTGCAAGTGTCTCCAGGAG	
+120/124	GGCTAAGTGGAGTCGGAAGG	48,711,497-48,715,805
	GGCCCCATTTCCCATCTCAC	
+185/191	GGGAAGCCCCCGAGTGCTGT	48,645,074-48,650,422
	GGTGCTCTCCCCTGCAGCCG	
+18/21	GGGCTATATCCTGGGATGAC	48,815,241-48,818,255
	GGAGGGTTAGCTTAGACTTT	
+21/23	GGACCTTCCATTCACGCTAA	48,813,509-48,815,241
	GGGCTATATCCTGGGATGAC	
+23/29	GGGACACGTCTCATGTATAA	48,806,974-48,813,530
	GGACCTTCCATTCACGCTAA	
+29/31	GGGCCTGGTCTACTTCCCAT	48,805,350-48,806,976
	GGGACACGTCTCATGTATAA	
+31/32	GGTACCTCTTCACATGTGTA	48,804,389-48,805,356
	GGGCCTGGTCTACTTCCCAT	
1-338	GGGGGGGACACAGTTTGTCT	48,814,366-48,814,703
	GGCTGTTTGCCCGATGGATC	
339-730	GGCTGTTTGCCCGATGGATC	48,814,704-48,815,095
	GGAGTTTTGAGGGTAACTAA	
1-730	GGGGGGGACACAGTTTGTCT	48,814,366-48,815,095
	GGAGTTTTGAGGGTAACTAA	

Supplementary Table 2. Primers used for Runx1 ChIP-qPCR.

Primer name	Primer sequence
Zbtb16_A_F	AAGTCATCAGCGGAGTCTTCTC
Zbtb16_A_R	AAGTATCAGGCCAAGTGAGGTG
Zbtb16_B_F	ACCCAATAGTGCTGTGAGCTTC
Zbtb16_B_R	TCTGCCATGAAAGTGAGATGC
Zbtb16_C_F	CCTCTGCTCAGTGGTTAAGTCG
Zbtb16_C_R	AGATGCGTGGCCAAGATAGAG
Zbtb16_D_F	AGGTTTACACATCTCACCACAAGAC
Zbtb16_D_R	CGAGCTTTGAACAGTGGAGAG
Zbtb16_E'_F	GAGATGCCCAGACAAACTGTG
Zbtb16_E'_R	TGTGGTTGGATAAAGGAAGCTC
Zbtb16_E_F	TGAGCTTCCTTTATCCAACCAC
Zbtb16_E_R	AATGTCTGCAAACCGACAGTG
MyoD1_F	TGCCATTGAGAGGCAAAGTC
MyoD1_R	TATTCTCTGAGCCCTCTCATGC
Lekr1_F	GGCAAGCTGTACACTTTCAGG
Lekr1_R	AGGGCTAAGATGTAGGTCAGTGG
Ptprc_1_F	TCTTCTCCTCGCACACTTCTG
Ptprc_1_R	GTTGCACCATTTGGAAGCTC
Ptprc_2_F	AAAGACAGGCCAATCCTTCC
Ptprc_2_R	ACCCGTGGCTTTGTATACTGTG
Gapdh_F	TCGTCTCTGAACCCTCTTTCC
Gapdh_R	CAAATCAGCCTCCCTTCTCC
Amhr2_F	ATTCTCTGCTCCTCCCTTTCTC
Amhr2_R	TCTGTCCTATCCCGGTCTCTG
β-globin_F	GCCATCGTTAAAGGCAGTTATCA
β-globin_R	TGCTATCATGGGTAATGCCAAA
