# Supplementary material for: A shared Runx1-bound Zbtb16 enhancer directs innate and innate-like lymphoid lineage development
Source: Nat Commun. 2017 Oct 16;8:863. doi: 10.1038/s41467-017-00882-0 (PMC5643357; doi:10.1038/s41467-017-00882-0)
Supplement: Supplementary file 2 — Peer Review File [file 41467_2017_882_MOESM2_ESM.pdf]

Editorial Note: this manuscript has been previously reviewed at another journal that is not operating a transparent peer review scheme. This document only contains reviewer comments and rebuttal letters for versions considered at Nature Communications.

REVIEWERS' COMMENTS:

Reviewer #1 (Remarks to the Autho

The authors have faithfully made responses to the majority of the reviewers' comments.
However, there is one issue to be addressed. Although the authors showed the Ab23980 antibody specificity by IP assay in Fig. 1, they should show its specificity by Chip assay. Therefore, to show the specificity of Ab23980 antibody, the motif analysis of Runx1 binding peaks using their Runx1 ChIP-seq data is recommended to carry out. This is more informative for the Runx1 binding to the Runx-binding sequence in the enhancer regions.
This reviewer recommends to publish the revised version after this minor revision.

Response to reviewer 1:

We performed the motif analysis and confirmed that the top motif for Runx1 ChiP-seq peaks was indeed a Runx motif.
